# Inter-relationships of depression and anxiety symptoms among widowed and non-widowed older adults: findings from the Chinese Longitudinal Healthy Longevity Survey based on network analysis and propensity score matching

**DOI:** 10.3389/fpubh.2025.1495284

**Published:** 2025-03-12

**Authors:** Yinglin Li, Doudou Lin, Xuan Gong, Dou Fu, Ling Zhao, Weibing Chen, Jie Chen, Shanshan Liu, Guirong Yang, Zhongxiang Cai

**Affiliations:** ^1^Department of Nursing, Renmin Hospital of Wuhan University, Wuhan, China; ^2^Department of Geriatrics, Renmin Hospital of Wuhan University, Wuhan, China; ^3^Department of Psychiatry, Renmin Hospital of Wuhan University, Wuhan, China; ^4^Florida State University College of Nursing, Tallahassee, FL, United States; ^5^Hubei Polytechnic Institute, Xiaogan, China

**Keywords:** older adults, widowed, depression, anxiety, network analysis

## Abstract

**Background:**

Depression and anxiety are prevalent mental health issues among older adult widowed adults. However, the symptom-level relationships between these conditions remain unclear. Due to the high correlations and complex relationships among various symptoms, this study employs network analysis to explore differences in the network structures of depression and anxiety symptoms between widowed and non-widowed older adults.

**Methods:**

Propensity score matching was used to identify widowed older adults with similar demographic characteristics. Data from 1,736 widowed and 1,736 matched controls were analyzed using the Chinese Longitudinal Healthy Longevity Survey (2017–2018). Depression and anxiety were measured by the Center for Epidemiologic Studies Depression Scale-10 (CESD-10) and the seven-item Generalized Anxiety Disorder Scale (GAD-7), respectively. Central and bridge symptoms were evaluated using expected influence (EI) and bridge expected influence (BEI), respectively.

**Results:**

Network analysis revealed similarities in central symptoms between widowed and non-widowed older adults, with both groups exhibiting “Feeling depressed or down” (CESD3), “Feeling tense and having difficulty relaxing” (GAD4), and “Being unable to stop or control worrying” (GAD2) as core symptoms. However, differences emerged in bridge symptoms. In the widowed group, “Feeling anxious, worried, or distressed” (GAD1) was most strongly connected to “Felt lonely” (CESD8); “Worrying too much about various things” (GAD3) was strongly linked to “Feeling increasingly exhausted and useless with age” (CESD4); and “Feeling depressed or down” (CESD3) had a strong association with “Becoming easily annoyed or irritable” (GAD6). In the non-widowed group, “Feeling anxious, worried, or distressed” (GAD1) exhibited the strongest association with “Having good sleep quality” (CESD10); “Getting upset over small matters” (CESD1) was closely connected to “Feeling anxious, worried, or distressed” (GAD1); and “Worrying too much about various things” (GAD3) was most strongly connected to “Feeling depressed or down” (CESD3).

**Conclusion:**

Common central and bridge symptoms highlight universal intervention targets. Addressing “Feeling depressed or down” in widowed and “Getting upset over small matters” in non-widowed older adults may help prevent depression-anxiety comorbidity. These findings support targeted interventions to improve mental health outcomes. Future research should evaluate tailored intervention effectiveness.

## 1 Introduction

The global population is experiencing an accelerating trend of aging. According to data from the World Health Organization, the global population aged 60 and above reached one billion in 2020 and is projected to rise to two billion by 2050 (1). Findings from China’s seventh national census indicate that the population aged 65 and above stands at 190.64 million, accounting for 13.5% of the total population (2). By 2040, the proportion of older adults in China is projected to reach 28% (3). Against this backdrop, the physical and mental health issues associated with aging are gaining increasing attention.

Widowhood significantly impacts the health of older adults, and with the aging population, the number of widowed older adults is on the rise (4). Approximately 47.48 million older adults in China, representing 26.89% of the older adult population, are widowed (5). The Conservation of Resources Theory highlights that widowhood is one of the most challenging life transitions, requiring more time and energy for adjustment compared to divorce (6). This transition involves not only the loss of a spouse but also severe psychological distress and increased vulnerability to various health issues (7).

Research indicates that older adults who have lost a spouse often face significant mental health challenges, including depression, anxiety, loneliness, and social withdrawal (8, 9). Following widowhood, social support tends to diminish (10), leading to lower subjective well-being compared to non-widowed older adults (11). Additionally, their physical health declines markedly (12), and they face an increased risk of cardiovascular diseases (13). The mortality risk for widowed individuals is 1.25 times higher than that for their non-widowed counterparts (14), and the risk of suicide is significantly elevated (15). These factors collectively contribute to the psychological distress experienced by widowed older adults. A systematic review and meta-analysis estimated the prevalence of depression within 1 month of widowhood to be 38.2% (21.9–55.8%) (16). Furthermore, widowed individuals are more likely to exhibit somatic and phobic anxiety symptoms (17), and experience higher levels of depression and anxiety compared to their non-widowed peers (18, 19).

The issue of depression and anxiety among older adults is complex. Depression and anxiety are the most prevalent mental health issues in older adults, significantly contributing to the global burden of disease (20, 21). Depression in older adults is characterized by persistent low mood, often accompanied by cognitive impairments and somatic symptoms (22). Globally, an estimated 280 million people suffer from depression, with older adults comprising 6.5% of this total (23). A systematic review and meta-analysis of 55 studies (*N* = 59,851) found a global prevalence of depression among older adults to be 35.1% (95% CI: 30.2–40.4%) (22). In China, 4.46% of older adults experience depression, while 35.19% experience depressive symptoms (24). Depression in later life can negatively impact physical health, daily functioning, and overall well-being. Depressive symptoms and major depressive disorder are frequently associated with chronic physical illnesses, such as stroke, diabetes, chronic obstructive pulmonary disease, cancer, Alzheimer’s disease, and arthritis (25, 26). Severe depression can even lead to disability, suicide, and increased mortality risk (27). Anxiety, characterized by excessive worry and nervousness, also severely impacts older adults. Studies indicate a prevalence of generalized anxiety disorder ranging from 0.2 to 32.2% among older adults in low- and middle-income countries (28). In China, the detection rate of anxiety in older adults ranges from 1.39 to 32.74% (19, 29). Anxiety is closely linked to the development of vascular dementia and cardiovascular diseases, profoundly impacting the quality of life for older individuals (30).

Depression and anxiety commonly co-occur (30), with comorbid anxiety disorders affecting 40 to 60% of individuals diagnosed with major depressive disorder (31). Older adults experiencing both depression and anxiety face increased healthcare burdens, elevated disability risks (32), and higher susceptibility to chronic illnesses compared to those with either condition alone (33, 34). They also report heightened psychological distress and more somatic symptoms such as chest discomfort, palpitations, and gastrointestinal issues. Prolonged stress-induced physiological and psychological hyperarousal can adversely affect bodily systems through hormonal and immune mechanisms, particularly impacting frail older adults (33), and ultimately leading to a significant decline in overall well-being (35).

As the high correlations and complex relationships among depression and anxiety, network analysis methods are indispensable for unraveling the complexities of depression and anxiety among older adults. Traditional psychopathology theories often rely on aggregated scores from standardized tests to gauge the severity of psychiatric symptoms (36). However, this approach overlooks the interconnectedness and unique characteristics of individual symptoms (37). Network analysis fills this gap by investigating the intricate and dynamic relationships between psychiatric symptoms (38). This methodology constructs a network where symptoms are nodes connected by edges, identifying core symptoms through metrics such as centrality, proximity, and strength. By emphasizing pivotal symptoms within the network, network analysis offers insights into the underlying mechanisms of mental illness. Currently, network analysis is widely utilized to study depression and anxiety symptoms across diverse populations, including adolescents (39, 40), college students (41, 42), individuals with chronic diseases (43, 44), disabled older adults (45), and medical professionals (46). These studies consistently demonstrate the interconnected nature of depression and anxiety symptoms. Network analysis not only identifies central symptoms within these networks but also underscores the significance of specific symptoms in shaping the overall symptom structure.

However, despite the growing body of research using network analysis to understand depression and anxiety, a critical knowledge gap remains: the comparative network structures of comorbid depression and anxiety symptoms in widowed versus non-widowed older adults have been largely unexplored. While one study examined depression symptom networks in these populations (47), it did not address the crucial interplay between depression and anxiety, which are known to be highly comorbid, especially in older adults (48, 49). Furthermore, that previous study did not utilize methods to control for baseline differences between the groups, potentially confounding the results. This is a significant limitation, as widowed and non-widowed older adults often differ on key demographic and health-related variables that could independently influence mental health outcomes.

To address this gap, the current study employed a rigorous methodology combining propensity score matching (PSM) and network analysis. PSM was used to create comparable widowed and non-widowed groups, minimizing selection bias by balancing key demographic and health-related confounders before comparing their depression and anxiety symptom networks. Utilizing data from the Chinese Longitudinal Healthy Longevity Survey (CLHLS), this study aims to identify central symptoms within each group and bridge symptoms linking depression and anxiety. By pinpointing these bridge symptoms, this study provides evidence for targeted, cost-effective interventions to prevent depression–anxiety comorbidity, reducing disease burden and healthcare costs in older adults.

## 2 Methods

### 2.1 Participants and procedures

This study utilizes data from the 2017–2018 wave of the CLHLS. The CLHLS is a longitudinal survey organized by the Center for Healthy Aging and Development Research at Peking University. The CLHLS is a major national research project in China aimed at understanding the multifaceted aspects of healthy aging. Initiated in 1998, it tracks a large cohort of older adults, collecting comprehensive data on a wide range of factors, including demographics, socioeconomic status, family structure, lifestyle behaviors (e.g., diet, exercise), health status (physical and mental), cognitive function, and access to healthcare. Following a baseline survey in 1998, the CLHLS has been conducted in seven waves (2000, 2002, 2005, 2008–2009, 2011–2012, 2014, and 2017–2018) across 23 provinces in China (50). To ensure a representative sample, the CLHLS employs a multistage, disproportionate, and targeted random sampling method, focusing on older adults aged 65 and above. Ethical approval for the CLHLS was obtained from the Peking University Biomedical Ethics Committee (IRB00001052-13074) and the Duke University Institutional Review Board (Pro00062871). The deidentified 2017–2018 CLHLS dataset used in this study is publicly available and accessible.

The inclusion criteria for this study were as follows: (1) participants aged 65 years or older; and (2) availability of complete basic demographic data, CESD-10, and GAD-7 scores (Supplementary Figure S1).

### 2.2 Measures

Sociodemographic data included age, education level, gender, current residence, current living arrangement, medical payer, and number of children. Sleep duration was included as a key covariate due to its well-documented bidirectional relationship with depression and anxiety (51), particularly in older adults (52, 53). Research indicates a significant positive correlation between sleep disturbances and depressive symptoms, with approximately 75% of individuals with depression experiencing sleep problems (54). Moreover, poor sleep quality substantially increases the risk of anxiety symptoms. Additionally, widowhood itself may disrupt sleep (55, 56), further contributing to mental health vulnerabilities in this population.

Depressive symptoms were assessed by using the Center for Epidemiologic Studies Depression Scale-10 (CESD-10), a validated instrument for Chinese older adults (57, 58). The CESD-10 comprises 10 items rated on a 4-point Likert scale (0 = ‘never’ to 3 = ‘always’), yielding a total score range of 0 to 30. Scores ≥10 indicate the presence of depressive symptoms, while scores ≥20 indicate severe depressive symptoms (59). The CESD-10 demonstrated good internal consistency (Cronbach’s alpha = 0.78).

Anxiety symptoms were evaluated by using the Seven-item Generalized Anxiety Disorder Scale (GAD-7), designed to assess the frequency of anxiety symptoms experienced over the past 2 weeks. The GAD-7 employs a 4-point Likert scale with seven items, ranging from 0 (not at all) to 3 (almost daily). Total scores range from 0 to 21, with higher scores indicating greater severity of anxiety symptoms (60). Threshold scores of 5, 10, and 15 denote mild, moderate, and severe levels of anxiety, respectively (60, 61). In this study, the GAD-7 demonstrated excellent internal consistency (Cronbach’s alpha = 0.92).

### 2.3 Statistical analysis

#### 2.3.1 Propensity score matching (PSM) and univariate analysis

To minimize demographic discrepancies between widowed and non-widowed groups of older adults, this study employed PSM utilizing the MatchIt package (version 4.5.1) (62) in R (version 4.3.1). The nearest neighbor method was implemented in a 1:1 ratio with a caliper of 0.05 (63). PSM serves to reduce selection bias in observational studies and ensure balance across study groups (63, 64). Propensity scores were computed via logistic regression models, with covariates including age, education level, sleep duration each day, gender, current living arrangement, medical payers, and number of children. Non-widowed older adults were selected to match the propensity scores of widowed counterparts, forming a balanced sample. Matching quality was evaluated by using standardized mean differences (SMDs), with values <0.10 indicating effective balance (65). Histograms were employed to visualize the propensity score distributions for both widowed and non-widowed groups before and after matching.

#### 2.3.2 Network analysis

Network analysis was conducted using R (version 4.3.1). The qgraph package (version 4.2.3) (66) and bootnet package (version 1.4.3) (67) were utilized for network visualization and estimation. Given that both the CESD-10 and GAD-7 utilize Likert scales, Spearman correlation coefficients were computed to estimate edges (68). The Elastic Net Least Absolute Shrinkage and Selection Operator (ELASSO) was employed to enhance the graph by assessing edge importance and minimizing spurious edges (69). The Extended Bayesian Information Criterion (EBIC) guided model selection, with a tuning parameter (𝛾 = 0.5) controlling sparsity (70). To enhance the visualization of the network, the edge weight threshold was set to 0.05.In the resulting network, each item is represented as a node, and pairwise associations between items are depicted as edges. Thicker edges indicate stronger correlations, with purple and red denoting positive and negative correlations, respectively.

Centrality indices quantitatively evaluate the structural significance of nodes within a network, identifying the most influential ones. These indices include strength, closeness, betweenness, and expected influence (EI). According to prior studies (68), EI represents the cumulative weights of edges connected to a node. In this study, EI was utilized to assess network centrality. Additionally, bridge expected influence (BEI) was computed to identify nodes bridging between communities of depression and anxiety symptoms, where higher BEI values increase the potential for activating interconnected communities in the network. These calculations were performed using the network tools package (version 4.2.2) (71). For visualization, EI and BEI are represented by z-score values. Furthermore, the mgm package (version 1.2) (72) was employed to estimate the predictability of each node, quantified by its R^2^ value. Node predictability indicates the extent to which a node can be predicted by its directly connected neighbors. Nodes with high predictability are more likely to be influenced effectively by interventions targeting their neighboring nodes.

The bootnet package (Version 1.4.3) (67) was utilized to evaluate both the accuracy and stability of the network analysis. Accuracy measures the extent to which sample estimates reflect true values, depicted by plotting 95% confidence intervals (CIs) of edge weights (nBoots = 1,500). Narrower CIs indicate higher accuracy. Stability was assessed using the correlation stability coefficient (CS-C) (nBoots = 4,000), where a CS of 0.70 denotes maximal acceptable sample reduction, and coefficients above 0.50 are generally acceptable, with a minimum of 0.25. Bootstrap tests of variance were employed to assess the stability of node EIs and edge weights, with a larger range in the black area indicating greater significance of differences.

To compare the differences in the network structure of depression and anxiety symptoms between the “widowed” and “non-widowed” groups, a network comparison test (NCT) was applied using the package “Network Comparison Test” (Version 2.2.2) (73).

## 3 Results

### 3.1 Study sample

A total of 4,982 widowed and 4,630 non-widowed older adults were screened for this study. 1,736 widowed older adults were matched to 1736 non-widowed older adults after PSM. Table 1 shows the demographic and clinical characteristics of the matched study sample. The SMD for the matched demographic variables was 0.03, indicating a well-balanced match. Supplementary Figure S2 shows the distribution of propensity scores and histograms.

**Table 1:** Characteristics of participants included in the study (*N* = 3,472).

Characteristics	Non-widowed (*N* = 1736)	Widowed (*N* = 1736)	t/χ^2^	*p*
	Mean ± SD or n (%)	Mean ± SD or n (%)		
Age (years)	82.86 ± 8.77	83.04 ± 9.56	−0.566	0.571
Education level (years)	3.68 ± 4.31	3.77 ± 4.26	−0.654	0.513
Sleep duration each day (hours)	7.27 ± 2.14	7.26 ± 2.24	0.170	0.865
Gender			0.195	0.659
Male	836 (48.2%)	822 (47.4%)		
Female	900 (51.8%)	914 (52.6%)		
Current residence			0.641	0.423
Urban	206 (11.9%)	190 (10.9%)		
Rural	1,530 (88.1%)	1,546 (89.1%)		
Current living arrangement			1.727	0.422
Family	1,495 (86.1%)	1,470 (84.7%)		
Living alone	179 (10.3%)	203 (11.7%)		
Nursing home	62 (3.6%)	63 (3.6%)		
Medical payers			6.44	0.490
Urban medical insurance	441 (25.4%)	455 (26.2%)		
Rural cooperative medical insurance	579 (33.4%)	578 (33.3%)		
Commercial health insurance	2 (0.1%)	2 (0.1%)		
No insurance	714 (41.2%)	701 (40.4%)		
Number of children	3.97 ± 1.89	3.95 ± 1.93	0.240	0.811
CESD-10 total score	9.46 ± 4.11	9.68 ± 4.11	−1.583	0.114
Depression symptoms (CESD-10 ≥ 10)	922 (53.1%)	969 (55.8%)	2.457	0.117
GAD-7 total score	1.58 ± 2.87	1.53 ± 2.85	0.475	0.635
Anxiety symptoms (GAD-7 ≥ 5)	234 (13.5%)	222 (12.8%)	0.305	0.580

CESD-10: The Center for Epidemiologic Studies Depression Scale-10; GAD-7: Seven-item generalized anxiety disorder scale.

### 3.2 Network structures in widowed versus non-widowed groups

#### 3.2.1 Network structure and centrality symptoms

**Figure 1:**
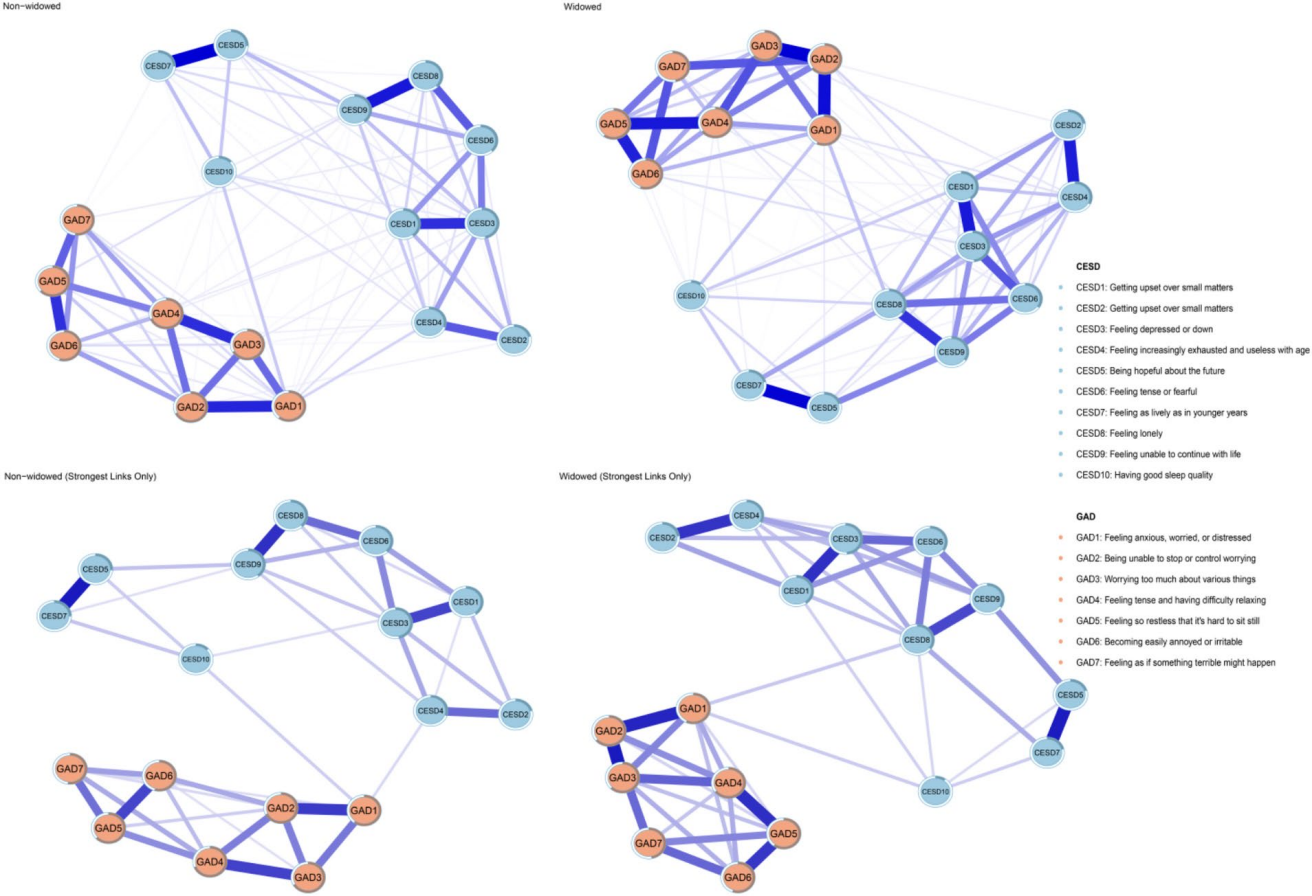
The network structure of depression and anxiety in widowed and non-widowed groups.

**Figure 2:**
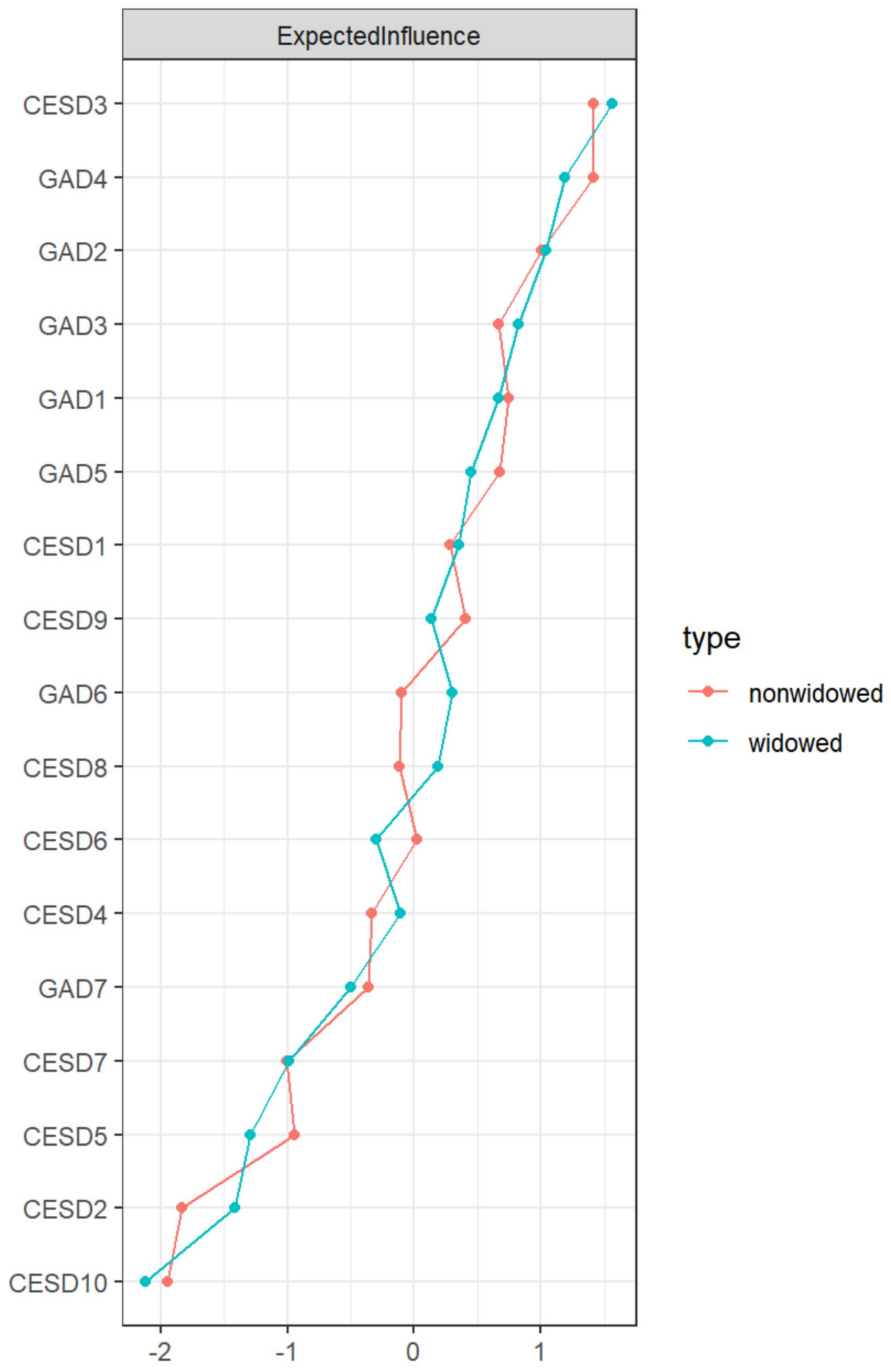
Standardized values (z-score) of expected influence (EI) for each node in the widowed and non-widowed groups.

Figure 1 illustrates the network structures of depression and anxiety symptoms in the widowed and non-widowed groups, including both the original networks and the networks with the weakest links removed (edge weight threshold set to 0.05). In the “non-widowed” group network, 93 out of 136 edges (68.4%) had non-zero weights, while in the “widowed” group network, 88 out of 136 edges (64.7%) had non-zero weights, indicating dense connectivity in both networks. Supplementary Table S1 provides the detailed edge weights for both groups. In the “widowed” group network, the most central symptoms were CESD3 (“Feeling depressed or down”; EI = 1.17), GAD4 (“Feeling tense and having difficulty relaxing”; EI = 1.09), and GAD2 (“Being unable to stop or control worrying”; EI = 1.06). Conversely, in the “non-widowed” group network, central symptoms included GAD4 (“Feeling tense and having difficulty relaxing”; EI = 1.13), CESD3 (“Feeling depressed or down”; EI = 1.13), and GAD2 (“Being unable to stop or control worrying”; EI = 1.05). Figure 2 presents a comparative visualization of node EIs between the widowed and non-widowed groups. The mean predictability was 0.418 for the widowed group and 0.442 for the non-widowed group. Notably, GAD4 in the widowed group (R^2^ = 0.648) and GAD4 in the non-widowed group (R^2^ = 0.691) exhibited the highest predictability. For detailed values of node EI, BEI, and predictability, refer to Supplementary Tables S2, S3.

#### 3.2.2 Bridge symptoms

**Figure 3:**
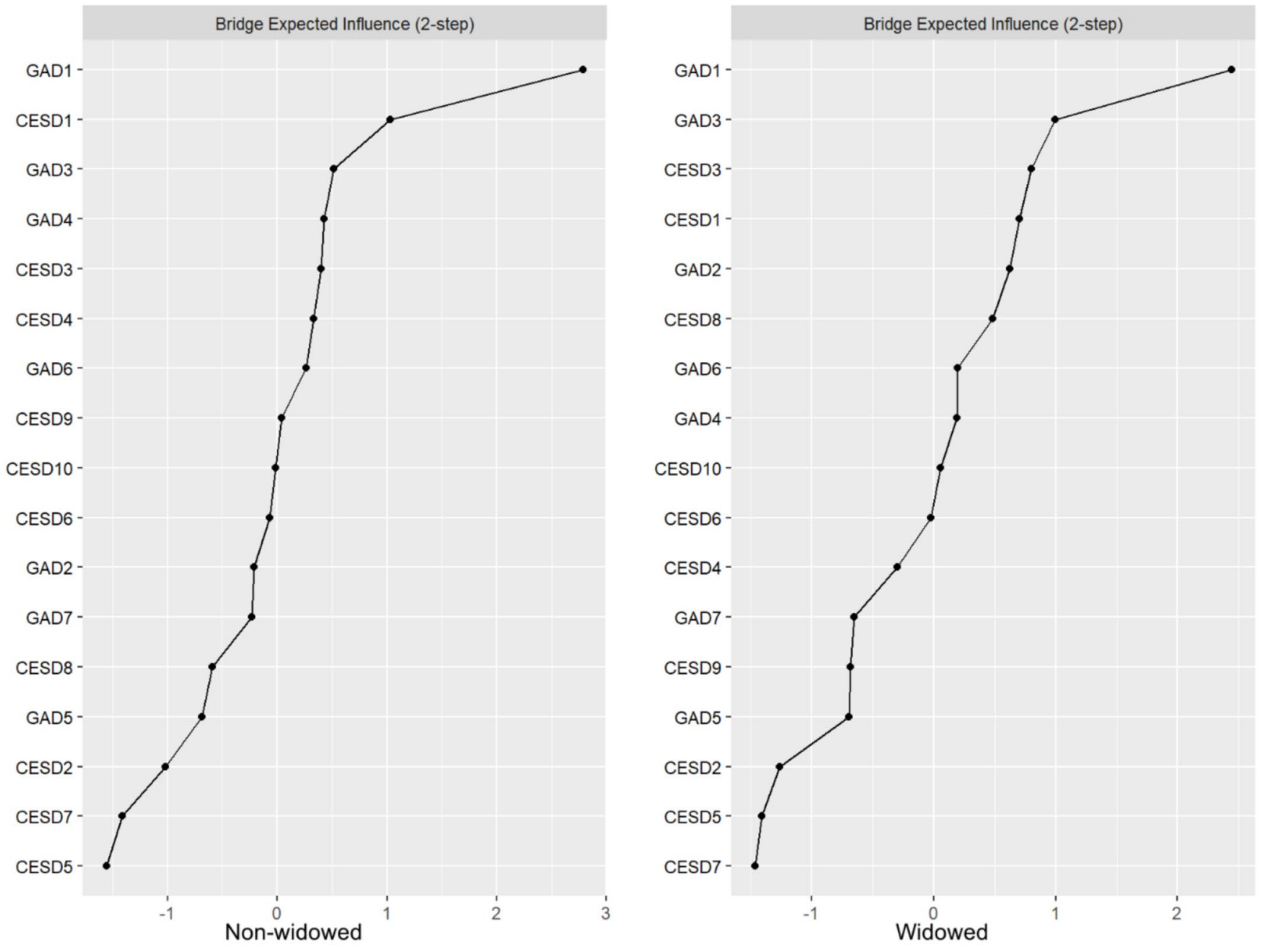
Bridge centrality indices (z-score) for the widowed and non-widowed groups.

Figure 3 illustrates the comparison of Bridge Expected Influence (BEI) between the widowed and non-widowed groups.

In the “widowed” group network, key bridge symptoms included GAD1 (“Feeling anxious, worried, or distressed”; BEI = 0.47), GAD3 (“Worrying too much about various things”; BEI: 0.32), and CESD3 (“Feeling depressed or down”; BEI = 0.30). Among them, GAD1 exhibited the strongest connection to CESD8 (“Felt lonely”) within the depression community (edge = 0.075); GAD3 showed the strongest connection to CESD4 (“Feeling increasingly exhausted and useless with age”) within the depression community (edge = 0.029); and CESD3 was most strongly linked to GAD6 (“Becoming easily annoyed or irritable “) within the anxiety community (edge = 0.040).

In the “non-widowed” group network, key bridging symptoms included GAD1 (“Feeling anxious, worried, or distressed”; BEI: 0.48), CESD1 (“Getting upset over small matters”; BEI: 0.30), and GAD3 (“Worrying too much about various things”; BEI: 0.25). Notably, GAD1 exhibited the strongest association with CESD10 (“Having good sleep quality”) within the depression community (edge = 0.080); CESD1 demonstrated the most robust connection with GAD1 (“Feeling anxious, worried, or distressed”) within the anxiety community (edge = 0.030); and GAD3 was most strongly linked to CESD3 (“Feeling depressed or down”) within the depression context (edge = 0.031).

#### 3.2.3 Network accuracy and stability

Supplementary Figure S3 displays narrow bootstrap 95% confidence intervals (CI) of estimated edge weights, indicating high accuracy. Supplementary Figure S4 illustrates the robust stability of EI and BEI. The CS coefficients for EI and BEI are 0.750 and 0.672 in both the widowed and non-widowed groups. The results of bootstrapped difference tests reveal statistically significant differences in most edge weights and node Expected Influence (EI), affirming the reliability of the principal findings (Supplementary Figure S5).

#### 3.2.4 Comparisons of the two network models

Based on the Network Comparison Test (NCT) results, comparisons between the network models of the non-widowed and widowed groups showed no significant differences in global strength (7.433 vs. 7.207, *S* = 0.227, *p* = 0.158) and network structure (M = 0.180, *p* = 0.050). There are significant differences in edge weights between the non-widowed and widowed groups in the network (*p* < 0.05).

## 4 Discussion

This study is among the first to employ network analysis and propensity score matching (PSM) to explore depression and anxiety symptom networks among widowed and non-widowed older adults in China. The results revealed no significant differences in the overall network structure and strength of depression and anxiety symptoms between the two groups. This suggests that widowhood, in itself, may not significantly alter the relationships between these symptoms or their overall severity. The central symptoms were consistent across both groups, including “Feeling depressed or down” (CESD3), “Feeling tense and having difficulty relaxing” (GAD4), and “Being unable to stop or control worrying” (GAD2). Common bridge symptoms for both groups were “Feeling anxious, worried, or distressed” (GAD1) and “Worrying too much about various things” (GAD3). However, “Feeling depressed or down” (CESD3) emerged as a unique bridge symptom for the widowed group, while “Getting upset over small matters” (CESD1) was specific to the non-widowed group.

### 4.1 Strengths of our study

The main strengths of this study include its large sample size and highly representative data from the latest wave of the CLHLS. The study used PSM and network analysis methods to explore the interaction between depression and anxiety symptoms in widowed older adults compared to demographically matched non-widowed older adults. The PSM approach minimized the influence of demographic imbalances between the widowed and non-widowed groups, resulting in more robust network analysis outcomes.

### 4.2 Similar network structures between groups

We found no significant differences in overall network strength and structure between widowed and non-widowed older adults, consistent with the findings of Xue et al. (74), but differing from those of Pan and Liu (47) reported certain differences in the depression networks of widowed and non-widowed older adults in China, which may be attributed to variations in datasets (CFPS) and methodological approaches. Our study employed propensity score matching (PSM) to minimize intergroup heterogeneity. The lack of data on the time elapsed since spousal loss in the CLHLS dataset may have attenuated the effects of recent widowhood, as prior research indicates that the impact of spousal loss is most pronounced within the first year (16), Moreover, common aging-related challenges, such as declining health and reduced autonomy, may contribute to similar symptom networks across groups (2), This aligns with the Conservation of Resources Theory (6), which posits that while both groups experience resource depletion (e.g., health, social networks), widowed individuals may compensate through alternative support systems.

In terms of network structure, both widowed and non-widowed older adult groups exhibited densely interconnected symptom networks, characterized by predominantly non-zero edge weights, indicating high comorbidity between anxiety and depression symptoms.

These findings aligned with previous research across various populations, including patients with COPD (43), disabled older adults (45), older adult individuals with hypertension (2), as well as other groups such as nurses (46), adolescents (39), and college students (41). These studies suggested that mental health issues were not isolated but were intertwined with multiple factors, forming complex network relationships (45).

### 4.3 Central symptom similarities

Network analysis revealed similar central symptoms of anxiety and depression in both widowed and non-widowed older adults. However, “Feeling depressed or down” (CESD3) exhibited slightly higher centrality in the widowed group than in the non-widowed group. “Feeling depressed or down,” as a negative emotion, is a core symptom required for the diagnosis of major depressive disorder (MDD) (31, 75). Widowhood precipitated significant life changes, including altered lifestyles, shifts in social roles, and increased financial strain (76, 77). From the perspective of attachment theory (78), the loss of a partner signifies the loss of a primary attachment figure, which could trigger intense separation anxiety and insecurity. This, in turn, impacted an individual’s self-worth and future outlook, rendering them more susceptible to feelings of sadness. This result is consistent with findings from other studies on older adults. For instance, network analyses of anxiety and depression in older adults with hypertension and disabilities have identified “Feeling depressed or down” as the most central symptom (2, 45). This may have been attributable to age-related declines in physical function and the exacerbation of pre-existing chronic conditions, both of which contributed to heightened depressive symptomatology.

In the non-widowed older adult group, “Feeling tense and having difficulty relaxing” (GAD4) exhibited marginally higher centrality. In China, sociocultural norms like filial piety, and legal frameworks such as the Marriage Law and the Law on the Protection of the Rights and Interests of Older Adults, established caregiving for older adults as a familial duty (79). Non-widowed older adults, particularly women, frequently undertook substantial caregiving responsibilities for spouses or other family members (80). Research indicated that caregiving demands substantial time, energy, and emotional resources, while simultaneously presenting caregivers with challenges such as the care recipient’s emotional lability and progressive decline in health (81). According to stress-coping theory (82), this prolonged exposure to high-stress caregiving can lead to physical and emotional exhaustion, hindering rest and relaxation and increasing the likelihood of anxiety symptoms, such as “Feeling tense and having difficulty relaxing” (GAD4). The higher centrality of GAD4 in this group may therefore reflected the significant caregiving burden experienced by many non-widowed older adults in China.

“Being unable to stop or control worrying” (GAD2) was significant core symptom in both groups, reflecting the pervasive uncertainty and anxiety frequently experienced by older adults in response to stressors such as aging, illness, and bereavement. Furthermore, “Feeling depressed or down” (CESD3) and “Being unable to stop or control worrying” (GAD2) were core symptoms for both groups. This aligns with the current perspective that these symptoms are central criteria and essential for diagnosing depression and generalized anxiety disorder (75, 83). This indicates that core symptoms of anxiety and depression are shared across different populations. Sadness and worry may represent common emotional responses to diverse life stressors and health challenges. From the perspective of cognitive-behavioral theory, negative cognitive patterns can exacerbate feelings of sadness and worry, potentially influencing other symptoms. Cognitive Behavioral Therapy (CBT) can be used to help older adults identify and change negative thought patterns and develop skills for managing stress and emotions, leading to better management of anxiety and depression symptoms (84, 85).

### 4.4 Different bridge symptoms between groups

The common bridging symptoms in both groups were “Feeling anxious, worried, or distressed” (GAD1) and “Worrying too much about various things” (GAD3). “Feeling depressed or down” (CESD3) and “Getting upset over small matters” (CESD1) were unique bridging symptoms for the widowed and non-widowed groups, respectively.

There were differences in bridging symptoms between the two groups. In the widowed group, “Feeling anxious, worried, or distressed” (GAD1) and “Felt lonely” (CESD8) were most closely connected. This suggested that in the widowed group, anxiety was closely linked with feelings of loneliness. The social isolation and emotional loss associated with widowhood may have exacerbated feelings of loneliness, making older adults more prone to depression. Previous research also indicated that widowed older adults experienced higher levels of loneliness (86–88). Studies employed the Dual-Process Bereavement Group Intervention-Chinese (DPBGI-C) had effectively reduced loneliness and anxiety in widowed older adults (89). “Worrying too much about various things” (GAD3) and “Feeling increasingly exhausted and useless with age” (CESD4) were closely connected, suggesting that excessive worrying in widowed older adults may have led to or exacerbated feelings of exhaustion and worthlessness. Widowed older adults often faced challenges such as loneliness, social isolation (76), and adverse health outcomes related to widowhood (90). The life changes and uncertainties brought about by widowhood led to increased worry about the future (91, 92). Cognitive appraisal theory suggests that an individual’s evaluation of events affects their emotional responses (93). Future research could further explore the relationship between widowed individuals’ cognitive appraisal of future uncertainties and their depressive emotions. “Feeling depressed or down” (CESD3) was most strongly linked to “Becoming easily annoyed or irritable” (GAD6) within the anxiety community, indicating a stronger association between depressive and irritability symptoms. The grieving process after widowhood was complex, and research shows that widowed older adults were more likely to experience “angry grief,” which was often directed towards the deceased partner, oneself, others, or even fate. This manifested as irritability and dissatisfaction with those around them (91). Techniques such as relaxation training and internet-based CBT may help widowed older adults better manage their emotions (94).

In the non-widowed group, “Feeling anxious, worried, or distressed” (GAD1) was most closely linked with “Having good sleep quality” (CESD10). This suggested that for non-widowed older adults, sleep quality may have been an important factor linking anxiety and depression. Previous research established the interrelationship between sleep, anxiety, and depression (95–98). For non-widowed older adults, particularly those with low marital satisfaction or those caring for chronically ill spouses, experienced significant psychological stress and life burden (99–101), potentially leading to increased anxiety, decreased sleep quality (102), and a heightened risk of depression. “Feeling anxious, worried, or distressed” may have reflected an overactive state of the nervous system, which could disrupt natural sleep rhythms and affect sleep quality. Simultaneously, insufficient sleep could exacerbate the overactivation of the nervous system, creating a vicious cycle that ultimately leads to the development of depressive symptoms (103). Interventions such as mindfulness-based stress reduction, mindfulness-based cognitive therapy, cognitive-behavioral therapy for insomnia, muscle endurance training, and Tai Chi have proven effective in improving insomnia and anxiety in older adults (104, 105). Furthermore, “Getting upset over small matters” (CESD1) was closely linked with “Feeling anxious, worried, or distressed” (GAD1), indicating heightened emotional sensitivity in this group. These individuals were prone to being affected by seemingly minor events in daily life, leading to exaggerated emotional responses. This finding aligns with previous research, which shows that high emotional sensitivity is often closely related to anxiety symptoms (106). “Worrying too much about various things” (GAD3) was most closely linked with “Feeling depressed or down” (CESD3), highlighting excessive worry as a key driver of depressive emotions in non-widowed older adults. These worries may have stemmed from concerns about aging, illness, role changes, and family health (4, 81, 107). Accessing professional psychological counseling and engaging in social activities can help build social support networks and foster a sense of purpose, potentially alleviating anxiety (4, 108).

### 4.5 Limitations

This study has several limitations. First, reliance on self-report questionnaires to assess depression and anxiety symptoms introduces potential response biases, such as social desirability and recall bias, particularly relevant given potential memory decline in older adults. Future research could mitigate this by incorporating multi-source data (interviews, observations) for a more comprehensive assessment. Second, the sample’s limitation to older adults in China restricts the generalizability of findings to other cultures and age groups. Cultural variations in widowhood coping mechanisms may influence symptom manifestation and network structure; cross-cultural comparisons are needed to explore these differences. Third, the network structures observed may be influenced by the specific measures used. Replicating these findings with different assessment instruments is crucial. Fourth, the cross-sectional design limits inferences about the temporal dynamics of depressive and anxiety symptoms. Longitudinal studies are needed to track symptom trajectories in older adults before and after widowhood, examining how these networks evolve to inform long-term psychological interventions. Finally, our reliance on the CLHLS dataset, primarily comprising individuals from rural areas, may limit the representativeness of urban older adults. Future research should include a more diverse sample, incorporating urban older adults, to enhance the generalizability of the findings.

## 5 Conclusion

In conclusion, our study is among the first to employ network analysis and propensity score matching (PSM) to explore depression and anxiety symptom networks among widowed and non-widowed older adults in China. While overall network structure and symptom intensity did not differ significantly between groups, crucial differences emerged in bridging symptoms. “Feeling depressed or down” (CESD3) played a more central role in the widowed group, potentially reflecting the emotional impact of bereavement, while “Getting upset over small matters” (CESD1) was more prominent in the non-widowed group, possibly linked to caregiving responsibilities. These findings offer valuable insights into the mental health of both widowed and non-widowed older adults and may inform the development of more targeted intervention strategies.

## Data Availability

The original contributions presented in the study are included in the article/Supplementary material, further inquiries can be directed to the corresponding author.
