# Supplementary material for: Inter-relationships of depression and anxiety symptoms among widowed and non-widowed older adults: findings from the Chinese Longitudinal Healthy Longevity Survey based on network analysis and propensity score matching
Source: Front Public Health. 2025 Mar 12;13:1495284. doi: 10.3389/fpubh.2025.1495284 (PMC11936798; doi:10.3389/fpubh.2025.1495284)
Supplement: Supplementary file 1 [file Supplementary_file_1.docx]

Supplementary Material

1. Table S1. Specific Edge Weights of the Network for the Widowed and Non-Widowed Groups
2. Table S2. Specific Node EI and BEI Values for the Network of the Widowed and Non-Widowed Groups
3. Table S3. Specific Predictability Values for the Network of the Widowed and Non-Widowed Groups
4. Figure S1. Flowchart of Sample Selection
5. Figure S2. Distribution and Histogram of Propensity Scores Before and After Matching for the "Widowed" and "Non-Widowed" Groups
6. Figure S3. Accuracy of Edge Weights
7. Figure S4. Network Stability of Node EI and BEI in the Widowed and Non-Widowed Groups
8. Figure S5. Differential Test of Node EI and Edge Weights

**Table S1. Specific Edge Weights of the Network for the Widowed and Non-Widowed Groups**

**Table S2. Specific Node EI and BEI Values for the Network of the Widowed and Non-Widowed Groups**

**Table S3. Specific Predictability Values for the Network of the Widowed and Non-Widowed Groups**

**Figure S1.** **Flowchart of sample selection**

People participate CLHLS 2017-2018 wave (N=15,874)

Participants aged 65 years old and above (N=15,779)

People with complete assessment of CESD-10, GAD-7 items and main demographic information (e.g., age, gender, marital status, education level, current residence, living arrangements, sleep duration each day, number of children and medical payers) (N=9,612)

PSM by age, education level, sleep duration each day, gender, current living arrangement, medical payers, and number of children with a ratio of 1:1 between the widowed group and the non-widowed group (N=3,472)

Non-widowed group (1,736)

Widowed group (N=1,736)

**Figure S2. Distribution and Histogram of Propensity Scores Before and After Matching for the "Widowed" and "Non-Widowed" Groups**


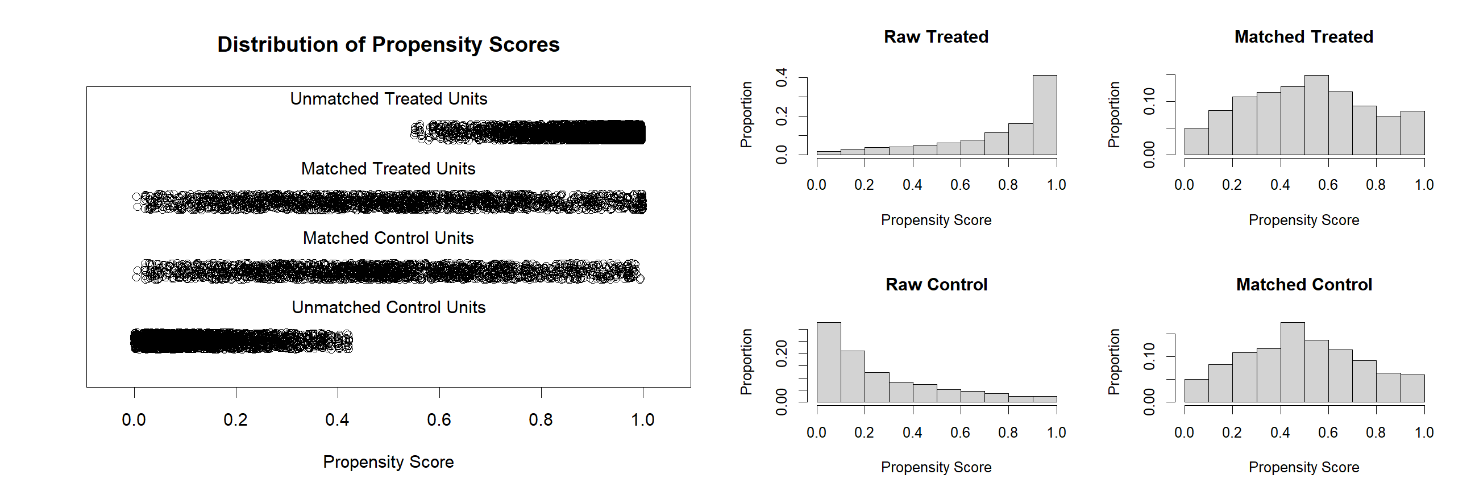


**Figure S3. Accuracy of Edge Weights**


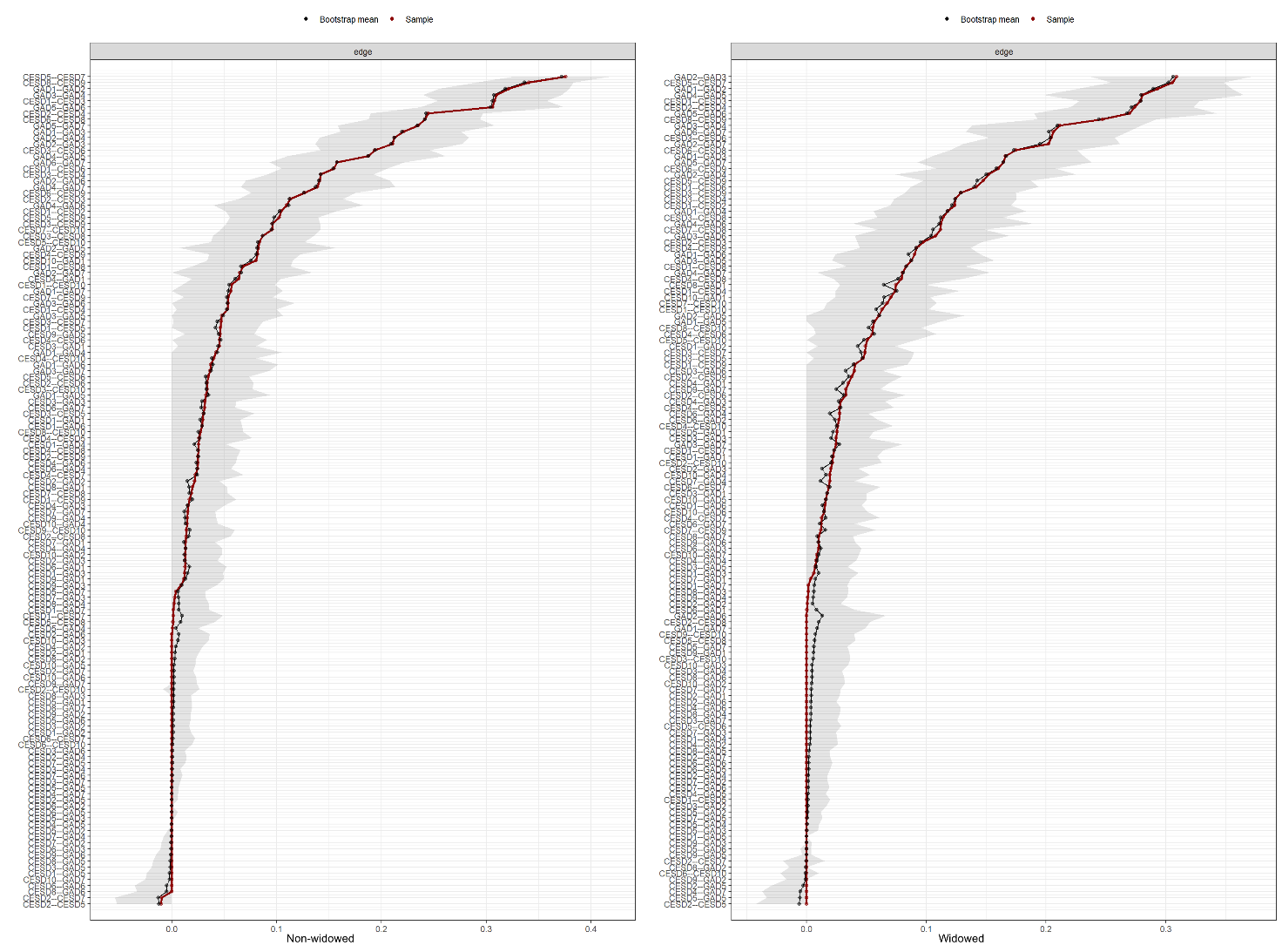


**Figure S4. Network Stability of Node EI and BEI in the Widowed and Non-Widowed Groups**


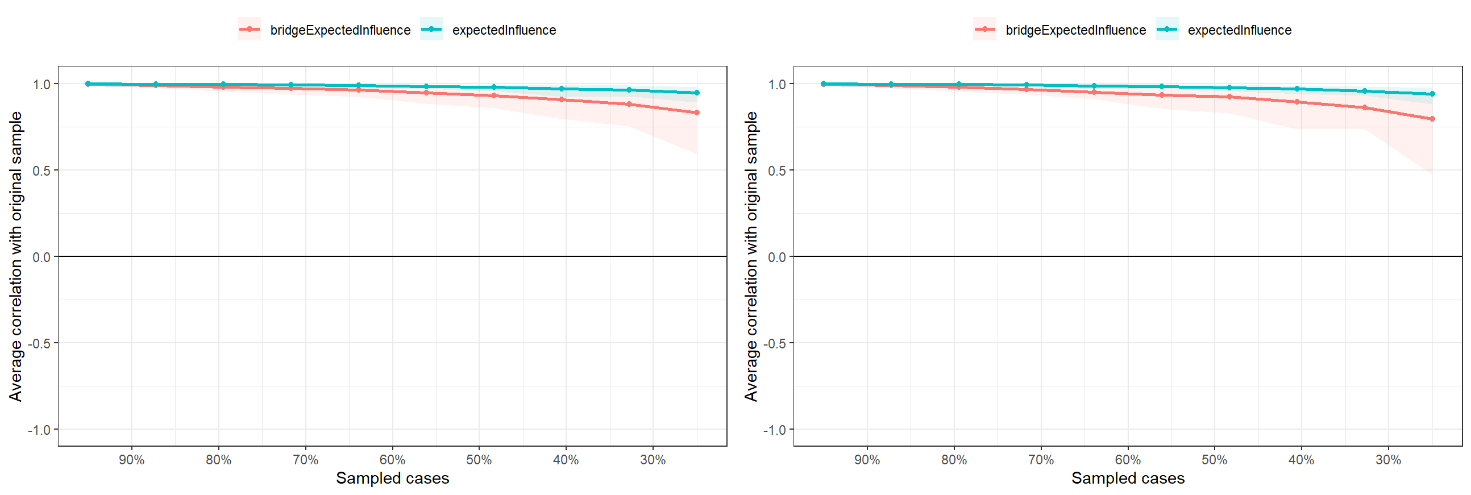


**Figure S5. Differential Test of Node EI and Edge Weights**

**
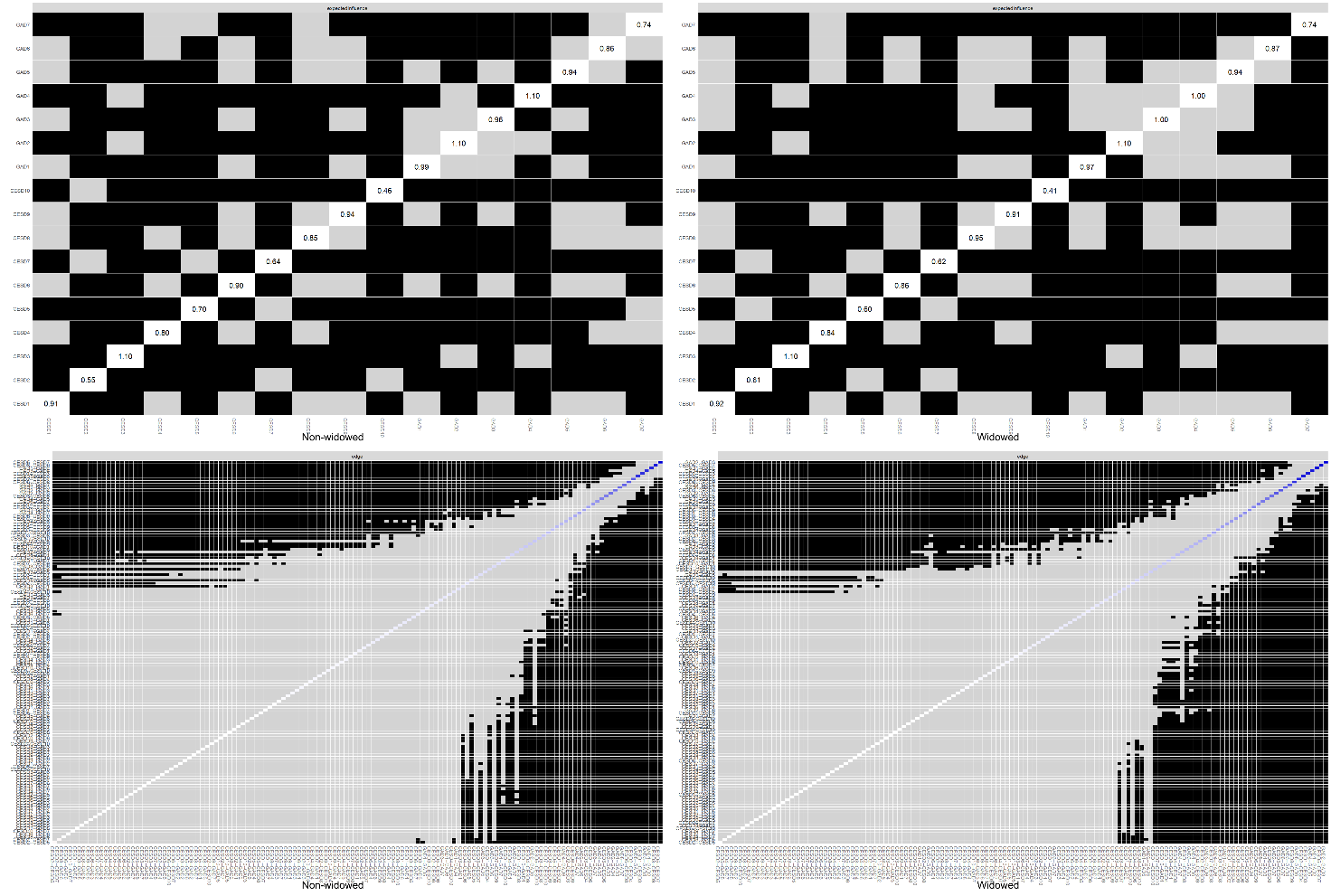
**
